# Simple method of fluid resuscitation in patients requiring emergency thoracotomy through direct cardiac cannulation

**DOI:** 10.1308/003588413X13511609957056d

**Published:** 2013-01

**Authors:** M Zakkar, I Hunt

**Affiliations:** St George’s Healthcare NHS Trust, UK

**Figure 1:**
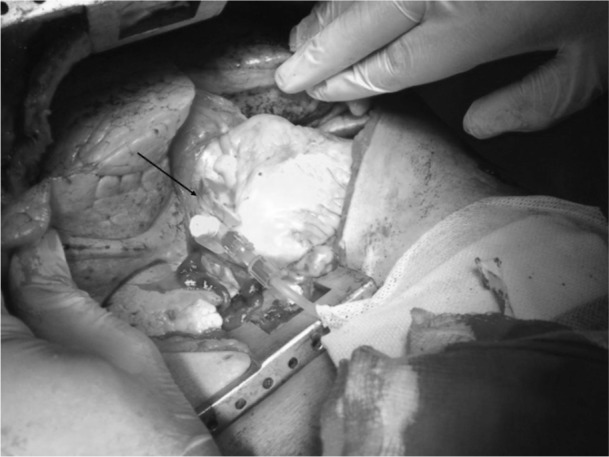
Clamshell thoracotomy with a large bore venous catheter in the right atrium (arrow)

Patients who are critically injured with imminent cardiac arrest may require immediate thoracotomy as an integral component of their initial resuscitation in the emergency department. Fluid resuscitation can be difficult owing to a shutdown state. A simple method for fluid delivery in patients requiring emergency department clamshell thoracotomy entails the insertion of a large bore venous catheter directly into the right atrium at its appendage and using it for fluid resuscitation (Fig 1). This method is quick, safe and easily reproducible. Once the patient is stable, the catheter can be removed and a simple purse string suture used to close the atrium.

